# [Bis(3-phenyl­prop-2-enyl­idene)propane-1,3-diamine-κ^2^ *N*,*N*′]dibromidocobalt(II)

**DOI:** 10.1107/S1600536808041408

**Published:** 2008-12-13

**Authors:** M. Montazerozohori, M. H. Habibi, M. Amirnasr, Siti Munirah Saharin, Hapipah Mohd Ali

**Affiliations:** a Department of Chemistry, Yasouj University, Yasouj, 75918-74831, Iran; b Catalysis Division, Department of Chemistry, University of Isfahan, Isfahan, 81745-73441, Iran; c Department of Chemistry, Isfahan University of Technology, Isfahan, 84156-83111, Iran; d Department of Chemistry, Faculty of Science, University of Malaya, 50603 Kuala Lumpur, Malaysia

## Abstract

In the crystal structure of the title compound, [CoBr_2_(C_21_H_22_N_2_)], the Co^II^ atom is four-coordinated by two bromide anions and two N atoms from the bidentate Schiff base ligand in a distorted tetra­hedral geometry.

## Related literature

For a related compound, see: Srivastava *et al.* (1990 ▶).
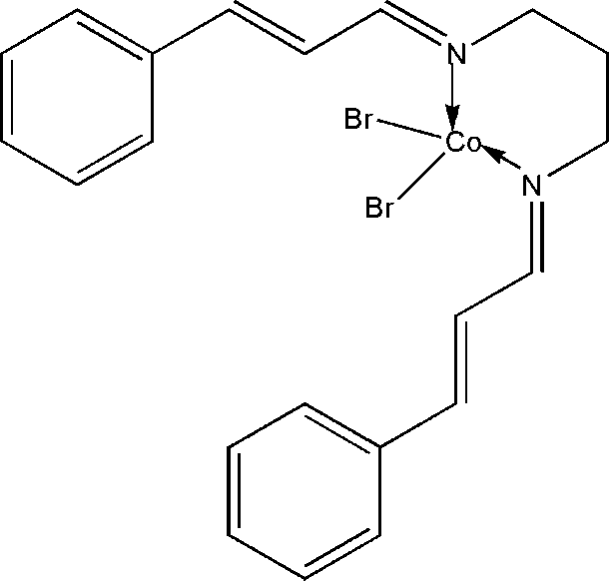


## Experimental

### 

#### Crystal data

[CoBr_2_(C_21_H_22_N_2_)]
*M* *_r_* = 521.16
Monoclinic, 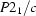

*a* = 14.0306 (19) Å
*b* = 11.9962 (16) Å
*c* = 13.6738 (19) Å
β = 110.375 (2)°
*V* = 2157.5 (5) Å^3^
*Z* = 4
Mo *K*α radiation
μ = 4.51 mm^−1^
*T* = 100 (2) K
0.32 × 0.12 × 0.10 mm

#### Data collection

Bruker APEXII area-detector diffractometer
Absorption correction: multi-scan (*SADABS*; Sheldrick, 1996 ▶) *T*_min_ = 0.323, *T*_max_ = 0.659
9901 measured reflections
3802 independent reflections
3275 reflections with *I* > 2σ(*I*)
*R*_int_ = 0.023

#### Refinement

*R*[*F* ^2^ > 2σ(*F* ^2^)] = 0.031
*wR*(*F* ^2^) = 0.080
*S* = 1.01
3802 reflections
235 parameters
H-atom parameters constrained
Δρ_max_ = 0.96 e Å^−3^
Δρ_min_ = −0.34 e Å^−3^

### 

Data collection: *APEX2* (Bruker, 2007 ▶); cell refinement: *SAINT* (Bruker, 2007 ▶); data reduction: *SAINT*; program(s) used to solve structure: *SHELXS97* (Sheldrick, 2008 ▶); program(s) used to refine structure: *SHELXL97* (Sheldrick, 2008 ▶); molecular graphics: *X-SEED* (Barbour, 2001 ▶); software used to prepare material for publication: *publCIF* (Westrip, 2009 ▶).

## Supplementary Material

Crystal structure: contains datablocks I, global. DOI: 10.1107/S1600536808041408/hg2447sup1.cif

Structure factors: contains datablocks I. DOI: 10.1107/S1600536808041408/hg2447Isup2.hkl

Additional supplementary materials:  crystallographic information; 3D view; checkCIF report

## Figures and Tables

**Fig. 1.**
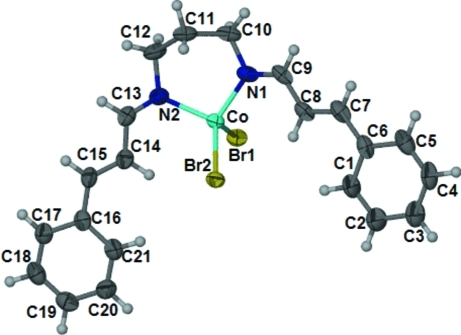
Thermal ellipsoid plot (Barbour, 2001) of the title complex at 70% probability level. Hydrogen atoms are drawn as spheres of arbitrary radius.

## supplementary crystallographic information

### Comment

Synthesis of a new four-coordinated complex of Co(II) is to identify the steric arrangement with this type of bidentate ligand and could lead to either pseudo-tetrahedral or square planar geometry. Reaction of anhydrous CoBr_2_ with the ligand, N,*N*-bis(3-phenyl-propenylidene)-1,3-propanediamine has yielded the title compound with distorted tetrahedral geometry.

### Experimental

The ligand of N,*N*-bis(3-phenyl-propenylidene)-1,3-propanediamine was prepared by condensation of 2?mmol of cinnamaldehyde and 1 mmol ethylenediamine in 10 ml dichloromethane. The mixture was cooled in an ice bath and then was added dropwise to a solution of 1 mmol anhydrous CoBr_2_ in 10 ml dichloromethane under nitrogen atmosphere. The mixture was stirred for 3 h and then filtered. To filtrate, 20 ml chloroform was added and kept overnight. The crystals suitable for X-ray were filtered off and washed with chloroform (64% yield). Elemental analysis for C_21_H_22_Br_2_CoN_2_ Calcd. C, 48.40; H, 4.25; N, 5.38%; Found: C, 48.32; H,4.21; N, 5.32%.

### Refinement

All H atoms were placed at calculated positions (C—H O.95 - 0.99Å) with U_iso_(H) set to 1.2 times U_eq_(C).

### Crystal data

**Table:**

[CoBr_2_(C_21_H_22_N_2_)]	*F*(000) = 1036
*M**_r_* = 521.16	*D*_x_ = 1.604 Mg m^−^^3^
Monoclinic, *P*2_1_/*c*	Mo *K*α radiation, λ = 0.71073 Å
Hall symbol: -P 2ybc	Cell parameters from 4346 reflections
*a* = 14.0306 (19) Å	θ = 2.3–29.6°
*b* = 11.9962 (16) Å	µ = 4.51 mm^−^^1^
*c* = 13.6738 (19) Å	*T* = 100 K
β = 110.375 (2)°	Block, green
*V* = 2157.5 (5) Å^3^	0.32 × 0.12 × 0.10 mm
*Z* = 4	

### Data collection

**Table:**

Bruker APEXII area-detector diffractometer	3802 independent reflections
Radiation source: fine-focus sealed tube	3275 reflections with *I* > 2σ(*I*)
graphite	*R*_int_ = 0.023
ω scan	θ_max_ = 25.0°, θ_min_ = 2.3°
Absorption correction: multi-scan (*SADABS*; Sheldrick, 1996)	*h* = −16→16
*T*_min_ = 0.323, *T*_max_ = 0.659	*k* = −14→14
9901 measured reflections	*l* = −15→16

### Refinement

**Table:**

Refinement on *F*^2^	Primary atom site location: structure-invariant direct methods
Least-squares matrix: full	Secondary atom site location: difference Fourier map
*R*[*F*^2^ > 2σ(*F*^2^)] = 0.031	Hydrogen site location: inferred from neighbouring sites
*wR*(*F*^2^) = 0.080	H-atom parameters constrained
*S* = 1.01	*w* = 1/[σ^2^(*F*_o_^2^) + (0.045*P*)^2^ + 2.376*P*] where *P* = (*F*_o_^2^ + 2*F*_c_^2^)/3
3802 reflections	(Δ/σ)_max_ < 0.001
235 parameters	Δρ_max_ = 0.96 e Å^−^^3^
0 restraints	Δρ_min_ = −0.34 e Å^−^^3^

### Special details

**Table:**

Geometry. All e.s.d.'s (except the e.s.d. in the dihedral angle between two l.s. planes) are estimated using the full covariance matrix. The cell e.s.d.'s are taken into account individually in the estimation of e.s.d.'s in distances, angles and torsion angles; correlations between e.s.d.'s in cell parameters are only used when they are defined by crystal symmetry. An approximate (isotropic) treatment of cell e.s.d.'s is used for estimating e.s.d.'s involving l.s. planes.
Refinement. Refinement of *F*^2^ against ALL reflections. The weighted *R*-factor *wR* and goodness of fit *S* are based on *F*^2^, conventional *R*-factors *R* are based on *F*, with *F* set to zero for negative *F*^2^. The threshold expression of *F*^2^ > σ(*F*^2^) is used only for calculating *R*-factors(gt) *etc*. and is not relevant to the choice of reflections for refinement. *R*-factors based on *F*^2^ are statistically about twice as large as those based on *F*, and *R*- factors based on ALL data will be even larger.

### Fractional atomic coordinates and isotropic or equivalent isotropic displacement parameters (Å^2^)

**Table:**

	*x*	*y*	*z*	*U*_iso_*/*U*_eq_	
Br1	0.43506 (3)	0.59326 (3)	0.13742 (3)	0.02898 (11)	
Br2	0.17275 (3)	0.75199 (3)	0.04140 (3)	0.02782 (11)	
Co	0.28730 (4)	0.63613 (4)	−0.00789 (3)	0.02382 (13)	
N1	0.3244 (2)	0.6980 (3)	−0.1274 (2)	0.0278 (7)	
N2	0.2176 (2)	0.4912 (2)	−0.0717 (2)	0.0286 (7)	
C1	0.3361 (4)	1.0847 (3)	0.0581 (3)	0.0456 (11)	
H1A	0.3094	1.0167	0.0732	0.055*	
C2	0.3418 (4)	1.1776 (4)	0.1210 (4)	0.0498 (11)	
H2A	0.3188	1.1725	0.1785	0.060*	
C3	0.3802 (3)	1.2761 (3)	0.1007 (4)	0.0433 (10)	
H3A	0.3846	1.3389	0.1444	0.052*	
C4	0.4120 (3)	1.2839 (3)	0.0176 (3)	0.0397 (10)	
H4A	0.4379	1.3527	0.0031	0.048*	
C5	0.4070 (3)	1.1931 (3)	−0.0459 (3)	0.0385 (10)	
H5A	0.4295	1.2000	−0.1035	0.046*	
C6	0.3690 (3)	1.0907 (3)	−0.0263 (3)	0.0327 (9)	
C7	0.3671 (3)	0.9943 (3)	−0.0910 (3)	0.0354 (9)	
H7A	0.3832	1.0071	−0.1521	0.042*	
C8	0.3451 (3)	0.8893 (3)	−0.0736 (3)	0.0304 (8)	
H8A	0.3283	0.8738	−0.0134	0.037*	
C9	0.3458 (3)	0.7994 (3)	−0.1420 (3)	0.0311 (8)	
H9A	0.3631	0.8156	−0.2017	0.037*	
C10	0.3299 (3)	0.6147 (3)	−0.2044 (3)	0.0329 (9)	
H10A	0.3895	0.5657	−0.1725	0.039*	
H10B	0.3389	0.6531	−0.2647	0.039*	
C11	0.2336 (3)	0.5444 (3)	−0.2418 (3)	0.0340 (9)	
H11A	0.1745	0.5939	−0.2520	0.041*	
H11B	0.2267	0.5121	−0.3106	0.041*	
C12	0.2293 (3)	0.4507 (3)	−0.1697 (3)	0.0375 (9)	
H12A	0.1714	0.4012	−0.2066	0.045*	
H12B	0.2924	0.4061	−0.1520	0.045*	
C13	0.1673 (3)	0.4295 (3)	−0.0322 (3)	0.0318 (8)	
H13A	0.1370	0.3643	−0.0696	0.038*	
C14	0.1526 (3)	0.4507 (3)	0.0653 (3)	0.0297 (8)	
H14A	0.1831	0.5143	0.1055	0.036*	
C15	0.0965 (3)	0.3817 (3)	0.0998 (3)	0.0313 (8)	
H15A	0.0666	0.3201	0.0563	0.038*	
C16	0.0764 (3)	0.3912 (3)	0.1974 (3)	0.0293 (8)	
C17	0.0274 (3)	0.3040 (3)	0.2283 (3)	0.0322 (8)	
H17A	0.0061	0.2406	0.1844	0.039*	
C18	0.0091 (3)	0.3078 (3)	0.3206 (3)	0.0354 (9)	
H18A	−0.0238	0.2471	0.3402	0.043*	
C19	0.0385 (3)	0.3995 (3)	0.3848 (3)	0.0364 (9)	
H19A	0.0262	0.4021	0.4488	0.044*	
C20	0.0866 (3)	0.4888 (4)	0.3552 (3)	0.0373 (9)	
H20A	0.1071	0.5521	0.3993	0.045*	
C21	0.1046 (3)	0.4853 (3)	0.2625 (3)	0.0347 (9)	
H21A	0.1362	0.5469	0.2424	0.042*	

### Atomic displacement parameters (Å^2^)

**Table:**

	*U*^11^	*U*^22^	*U*^33^	*U*^12^	*U*^13^	*U*^23^
Br1	0.0299 (2)	0.0322 (2)	0.02463 (19)	0.00440 (15)	0.00925 (15)	0.00136 (15)
Br2	0.0296 (2)	0.02873 (19)	0.0292 (2)	0.00210 (15)	0.01535 (15)	−0.00126 (15)
Co	0.0262 (3)	0.0260 (3)	0.0212 (2)	0.0021 (2)	0.0107 (2)	0.00126 (19)
N1	0.0265 (16)	0.0358 (18)	0.0228 (15)	0.0092 (14)	0.0106 (13)	0.0055 (13)
N2	0.0319 (16)	0.0307 (16)	0.0220 (15)	0.0019 (14)	0.0078 (13)	0.0000 (13)
C1	0.060 (3)	0.034 (2)	0.050 (3)	−0.014 (2)	0.028 (2)	0.004 (2)
C2	0.068 (3)	0.037 (2)	0.052 (3)	−0.012 (2)	0.030 (2)	−0.001 (2)
C3	0.041 (2)	0.032 (2)	0.054 (3)	−0.0029 (19)	0.013 (2)	0.005 (2)
C4	0.029 (2)	0.027 (2)	0.058 (3)	−0.0019 (17)	0.0083 (19)	0.012 (2)
C5	0.027 (2)	0.041 (2)	0.048 (2)	0.0038 (18)	0.0133 (18)	0.020 (2)
C6	0.0232 (19)	0.033 (2)	0.040 (2)	−0.0012 (16)	0.0086 (17)	0.0112 (17)
C7	0.030 (2)	0.041 (2)	0.038 (2)	0.0031 (18)	0.0145 (17)	0.0157 (18)
C8	0.0246 (18)	0.038 (2)	0.032 (2)	0.0037 (16)	0.0143 (16)	0.0099 (17)
C9	0.0229 (18)	0.043 (2)	0.030 (2)	0.0056 (17)	0.0127 (16)	0.0143 (18)
C10	0.034 (2)	0.043 (2)	0.0262 (19)	0.0143 (18)	0.0161 (17)	0.0061 (17)
C11	0.042 (2)	0.039 (2)	0.0235 (19)	0.0099 (19)	0.0144 (17)	−0.0010 (17)
C12	0.053 (3)	0.035 (2)	0.027 (2)	0.0027 (19)	0.0169 (19)	−0.0055 (17)
C13	0.034 (2)	0.0276 (19)	0.031 (2)	−0.0017 (17)	0.0072 (17)	−0.0023 (16)
C14	0.0291 (19)	0.0301 (19)	0.0258 (18)	−0.0031 (16)	0.0044 (15)	0.0005 (16)
C15	0.028 (2)	0.0303 (19)	0.032 (2)	−0.0077 (16)	0.0060 (16)	−0.0031 (16)
C16	0.0198 (18)	0.033 (2)	0.032 (2)	−0.0022 (16)	0.0039 (15)	0.0016 (16)
C17	0.029 (2)	0.028 (2)	0.038 (2)	−0.0001 (16)	0.0100 (17)	0.0014 (17)
C18	0.0249 (19)	0.036 (2)	0.046 (2)	0.0023 (17)	0.0140 (18)	0.0096 (19)
C19	0.030 (2)	0.047 (2)	0.035 (2)	0.0060 (19)	0.0141 (17)	0.0045 (19)
C20	0.032 (2)	0.041 (2)	0.040 (2)	−0.0026 (18)	0.0141 (18)	−0.0102 (18)
C21	0.030 (2)	0.035 (2)	0.039 (2)	−0.0036 (17)	0.0121 (17)	0.0011 (18)

### Geometric parameters (Å, °)

**Table:**

Br1—Co	2.3753 (6)	C10—C11	1.523 (5)
Br2—Co	2.3924 (6)	C10—H10A	0.9900
Co—N1	2.021 (3)	C10—H10B	0.9900
Co—N2	2.037 (3)	C11—C12	1.510 (5)
N1—C9	1.286 (5)	C11—H11A	0.9900
N1—C10	1.474 (5)	C11—H11B	0.9900
N2—C13	1.266 (5)	C12—H12A	0.9900
N2—C12	1.487 (4)	C12—H12B	0.9900
C1—C6	1.386 (5)	C13—C14	1.441 (5)
C1—C2	1.393 (6)	C13—H13A	0.9500
C1—H1A	0.9500	C14—C15	1.336 (5)
C2—C3	1.366 (6)	C14—H14A	0.9500
C2—H2A	0.9500	C15—C16	1.461 (5)
C3—C4	1.361 (6)	C15—H15A	0.9500
C3—H3A	0.9500	C16—C17	1.396 (5)
C4—C5	1.379 (6)	C16—C21	1.406 (5)
C4—H4A	0.9500	C17—C18	1.373 (5)
C5—C6	1.402 (5)	C17—H17A	0.9500
C5—H5A	0.9500	C18—C19	1.379 (6)
C6—C7	1.451 (6)	C18—H18A	0.9500
C7—C8	1.338 (5)	C19—C20	1.399 (6)
C7—H7A	0.9500	C19—H19A	0.9500
C8—C9	1.429 (5)	C20—C21	1.376 (5)
C8—H8A	0.9500	C20—H20A	0.9500
C9—H9A	0.9500	C21—H21A	0.9500
			
N1—Co—N2	100.80 (12)	N1—C10—H10B	109.5
N1—Co—Br1	111.12 (8)	C11—C10—H10B	109.5
N2—Co—Br1	108.83 (9)	H10A—C10—H10B	108.1
N1—Co—Br2	113.70 (8)	C12—C11—C10	115.2 (3)
N2—Co—Br2	110.32 (8)	C12—C11—H11A	108.5
Br1—Co—Br2	111.49 (2)	C10—C11—H11A	108.5
C9—N1—C10	117.3 (3)	C12—C11—H11B	108.5
C9—N1—Co	127.8 (3)	C10—C11—H11B	108.5
C10—N1—Co	114.9 (2)	H11A—C11—H11B	107.5
C13—N2—C12	116.6 (3)	N2—C12—C11	112.8 (3)
C13—N2—Co	124.9 (2)	N2—C12—H12A	109.0
C12—N2—Co	118.5 (2)	C11—C12—H12A	109.0
C6—C1—C2	120.6 (4)	N2—C12—H12B	109.0
C6—C1—H1A	119.7	C11—C12—H12B	109.0
C2—C1—H1A	119.7	H12A—C12—H12B	107.8
C3—C2—C1	120.6 (4)	N2—C13—C14	124.9 (3)
C3—C2—H2A	119.7	N2—C13—H13A	117.5
C1—C2—H2A	119.7	C14—C13—H13A	117.5
C4—C3—C2	119.7 (4)	C15—C14—C13	120.6 (3)
C4—C3—H3A	120.1	C15—C14—H14A	119.7
C2—C3—H3A	120.1	C13—C14—H14A	119.7
C3—C4—C5	120.7 (4)	C14—C15—C16	126.8 (3)
C3—C4—H4A	119.6	C14—C15—H15A	116.6
C5—C4—H4A	119.6	C16—C15—H15A	116.6
C4—C5—C6	120.8 (4)	C17—C16—C21	118.0 (3)
C4—C5—H5A	119.6	C17—C16—C15	119.3 (3)
C6—C5—H5A	119.6	C21—C16—C15	122.7 (3)
C1—C6—C5	117.6 (4)	C18—C17—C16	121.5 (4)
C1—C6—C7	121.8 (3)	C18—C17—H17A	119.3
C5—C6—C7	120.6 (3)	C16—C17—H17A	119.3
C8—C7—C6	126.7 (3)	C17—C18—C19	120.2 (4)
C8—C7—H7A	116.6	C17—C18—H18A	119.9
C6—C7—H7A	116.6	C19—C18—H18A	119.9
C7—C8—C9	122.6 (3)	C18—C19—C20	119.6 (4)
C7—C8—H8A	118.7	C18—C19—H19A	120.2
C9—C8—H8A	118.7	C20—C19—H19A	120.2
N1—C9—C8	123.9 (3)	C21—C20—C19	120.3 (4)
N1—C9—H9A	118.1	C21—C20—H20A	119.8
C8—C9—H9A	118.1	C19—C20—H20A	119.8
N1—C10—C11	110.7 (3)	C20—C21—C16	120.4 (4)
N1—C10—H10A	109.5	C20—C21—H21A	119.8
C11—C10—H10A	109.5	C16—C21—H21A	119.8
			
N2—Co—N1—C9	160.1 (3)	C10—N1—C9—C8	−179.2 (3)
Br1—Co—N1—C9	−84.6 (3)	Co—N1—C9—C8	−1.1 (5)
Br2—Co—N1—C9	42.1 (3)	C7—C8—C9—N1	−179.6 (4)
N2—Co—N1—C10	−21.7 (3)	C9—N1—C10—C11	−130.3 (3)
Br1—Co—N1—C10	93.5 (2)	Co—N1—C10—C11	51.3 (3)
Br2—Co—N1—C10	−139.7 (2)	N1—C10—C11—C12	−80.7 (4)
N1—Co—N2—C13	−169.1 (3)	C13—N2—C12—C11	147.5 (4)
Br1—Co—N2—C13	74.0 (3)	Co—N2—C12—C11	−35.0 (4)
Br2—Co—N2—C13	−48.7 (3)	C10—C11—C12—N2	70.0 (4)
N1—Co—N2—C12	13.6 (3)	C12—N2—C13—C14	176.2 (3)
Br1—Co—N2—C12	−103.3 (3)	Co—N2—C13—C14	−1.1 (5)
Br2—Co—N2—C12	134.1 (2)	N2—C13—C14—C15	178.7 (4)
C6—C1—C2—C3	−0.2 (7)	C13—C14—C15—C16	178.8 (4)
C1—C2—C3—C4	0.8 (7)	C14—C15—C16—C17	−171.5 (4)
C2—C3—C4—C5	−0.7 (6)	C14—C15—C16—C21	8.5 (6)
C3—C4—C5—C6	0.0 (6)	C21—C16—C17—C18	−1.7 (5)
C2—C1—C6—C5	−0.5 (6)	C15—C16—C17—C18	178.3 (3)
C2—C1—C6—C7	177.9 (4)	C16—C17—C18—C19	0.6 (6)
C4—C5—C6—C1	0.6 (6)	C17—C18—C19—C20	0.2 (6)
C4—C5—C6—C7	−177.8 (3)	C18—C19—C20—C21	0.0 (6)
C1—C6—C7—C8	−7.3 (6)	C19—C20—C21—C16	−1.2 (6)
C5—C6—C7—C8	171.1 (4)	C17—C16—C21—C20	1.9 (5)
C6—C7—C8—C9	−179.6 (4)	C15—C16—C21—C20	−178.0 (4)
